# Systematic Bibliometric Analysis of Research Hotspots and Trends on the Application of Virtual Reality in Nursing

**DOI:** 10.3389/fpubh.2022.906715

**Published:** 2022-05-19

**Authors:** Junqiang Zhao, Yi Lu, Fujun Zhou, Ruping Mao, Fangqin Fei

**Affiliations:** ^1^Department of Nursing, Xinxiang Medical University, Xinxiang, China; ^2^Department of Medical Engineering, Xinxiang Medical University, Xinxiang, China; ^3^Department of Children Rehabilitation, The First Affiliated Hospital of Xinxiang Medical University, Xinxiang, China; ^4^Department of Nursing, The Affiliated Eye Hospital of Nanjing Medical University, Nanjing, China; ^5^Department of Nursing, The First Affiliated Hospital of Huzhou University, Huzhou, China

**Keywords:** nursing, virtual reality, bibliometric, CiteSpace, VOS viewer

## Abstract

**Background:**

With the emergence of the metaverse, virtual reality, as a digital technology, must be getting hotter. High quality virtual reality related nursing knowledge scene learning is gradually replacing traditional education and intervention skills.

**Objective:**

This systematic study aimed to gain insights into the overall application of virtual reality technology in the study of nursing.

**Methods:**

Citations downloaded from the Web of Science Core Collection database for use in VR in nursing publications published from January 1, 2012, to December 31, 2021, were considered in the research. Information retrieval was analyzed using https://bibliometric.com/app, CiteSpace.5.8. R3, and VOS viewer.

**Results:**

A total of 408 institutions from 95 areas contributed to relevant publications, of which the United States is the most influential country in this research field. The clustering labels of cited documents were obtained from the citing documents. Virtual simulation, virtual learning, clinical skills, and dementia are the clustering labels of co-cited documents. The burst keywords represented the research frontiers in 2020–2021, which were knowledge and simulation.

**Conclusion:**

Virtual nursing has had an impact on both nurses and clients. With the emergence of the concept of the metaverse, the research and application of virtual reality technology in nursing will gradually increase.

## Introduction

Virtual reality (VR), also known as virtual simulation, is a completely synthetic world in which participants and observers can immerse themselves in and interact with (1). Mixed reality (MR) and Augmented Reality (AR) are subclasses of VR that are developed by VR technology (2, 3). Virtual reality can be utilized to generate a safe environment for activities (4); for example, it has many applications in the field of medicine. In many countries, virtual nursing is widely employed in nursing programs such as basic nursing, internal medicine and surgery, obstetrics, and pediatric nursing (5–8). According to the technology usage forecast of the United States in 2018, VR will experience the largest development in nursing in the next 5 years, and the adoption rate will increase to 45% from the current 10% (9). Previous studies have proven that VR can carry out effective nursing training or intervention in both disaster and community situations (10, 11). Besides, both home care and staff stress care based on VR technology have also been prove to be desirable in normal times (12–14).

This study aims to gain insight into the overall application of VR technology in nursing research through the following aspects: We analyzed Science Citation Index (SCI) papers for VR in nursing research using bibliometric methods. The citations of countries, regions, institutions, periodicals, study categories, keywords, and references were included in the data. Furthermore, as the core of this study, we established a visual and unbiased approach to exploring hotspot knowledge frontiers in the research area. The distribution and research influence of countries, regions, institutions, and journals are discussed using the research methods proposed in this study. The hotspots, future development space, and potential challenges of using virtual reality in nursing were all discussed. This provides references for computer researchers, nurses, educators, and experts in the field of medical engineering.

## Methods

### Selection of Citation Data

**Figure 1:**
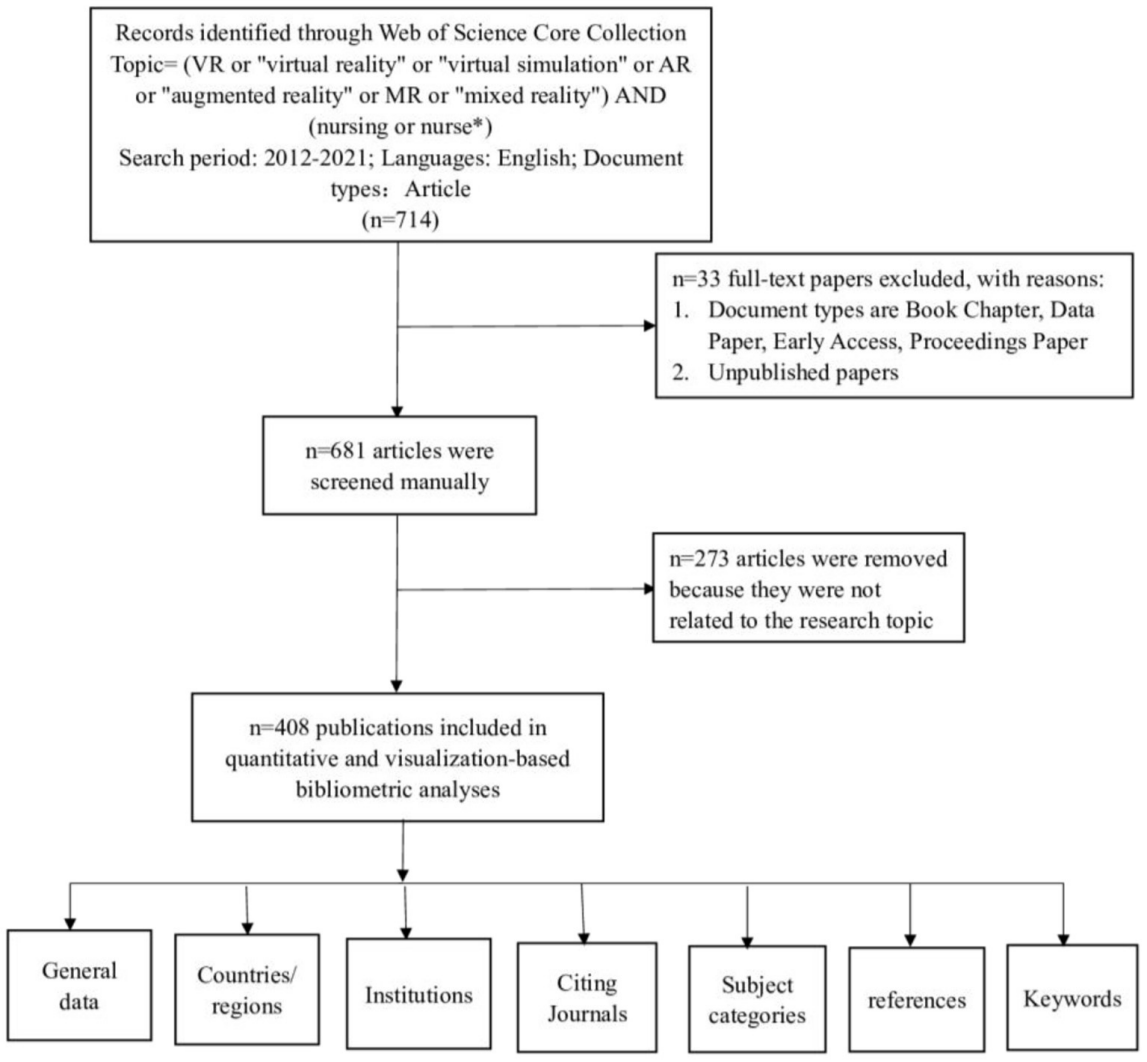
A frame flow diagram showing the detailed selection criteria and bibliometric analysis steps of applying VR to the study of nursing. Basic data of 408 literatures were included in the analysis.

On March 15, 2022, all citation data published between January 1, 2012, and December 31, 2021, were retrieved from the Web of Science Core Collection (WoSCC). They were independently verified by two authors (YL and WY). The search formula was TS= (VR or “virtual reality” or “virtual simulation” or AR or “augmented reality” or MR or “mixed reality”) and (nursing or nurse^*^). We selected English Literature articles, excluding book chapters, data papers, early access papers, and proceedings. In order to obtain more accurate results from the analysis, we manually removed publications unrelated to the application of VR in nursing. The following are the exclusion criteria: (1) The research topic is not virtual reality; (2) The research direction is unrelated to nursing; (3) The research is not a piece about the use of virtual reality in nursing. Finally, 408 documents remained. From each publication, we gathered the following basic data: title, publication year, country or region, institution, journal, references, and keywords. The detailed search and analysis processes are depicted in Figure 1.

### Statistical Analysis

Using https://bibliometric.com/app describes the situation in which documents are sent in different countries or areas. Vosviewer is being used to generate the heat of keywords. Cluster analysis of nations or regions, institutions, journals, research categories, keywords, and references using CiteSpace 5.8. CiteSpace program generates centrality. The WoSCC is used to calculate the H-index.

## Results

### Distribution of Articles by Publication Year

**Figure 2:**
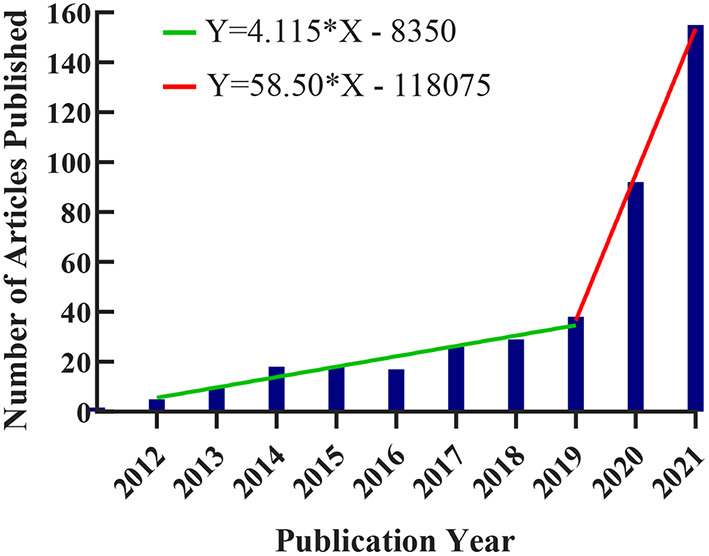
Trends in the number of publications on applying VR to the study of nursing from 2012 to 2021. The number of documents issued each year is represented by the blue bar. The green and red trend lines, respectively, represent the trend in which the number of published documents remains stable and the trend in which the number of published documents increases sharply.

As shown in Figure 2, the trend line formed by the number of annual publications has a small slope of 4.115 from 2012 to 2019. The increase in the number of papers published in was relatively gentle, with an average annual increase of <5. The number of literatures increased by 58.5 between 2020 and 2021. The number of published papers has increased dramatically in comparison to previous years.

### Countries or Regions

**Figure 3:**
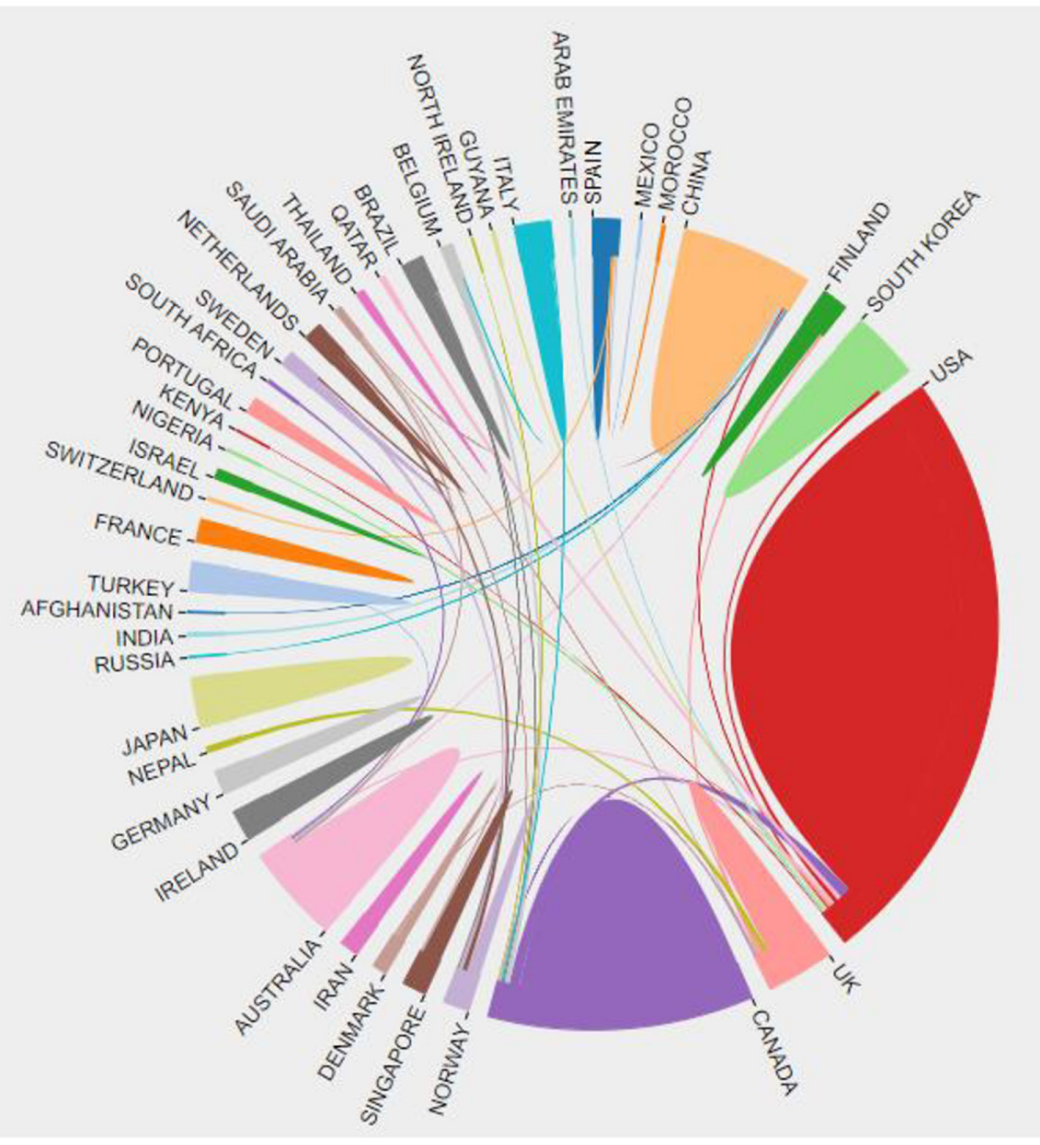
The cooperation of countries or regions that contributed to publications on applying VR to the study of nursing from 2012 to 2021. Different colored areas represent various countries. The size of the color block area describes the number of documents sent. The connection between different color blocks represents the countries' cooperative relationship.

**Figure 4:**
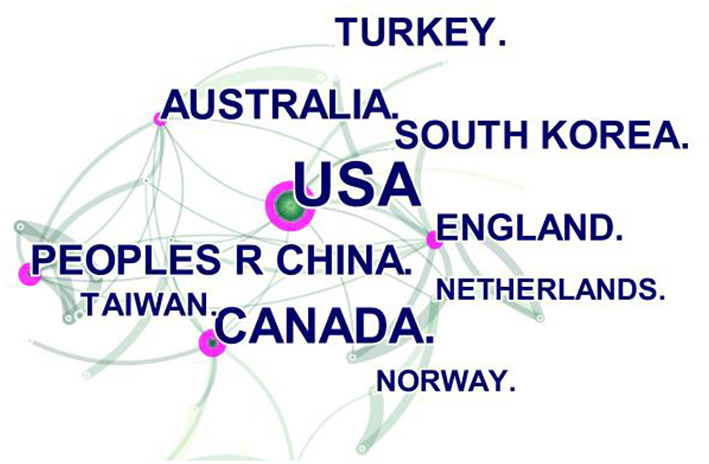
The cooperation of countries or regions that contributed to publications on applying VR to the study of nursing from 2012 to 2021. The number of documents sent by the country is represented by the size of the green node. Countries' cooperation is represented by connecting lines. The purple ring area size indicates the influence of the regional articles, which is equal to centrality.

A total of 95 nations or territories were mentioned in the citations. The color block area in Figure 3 represents the number of documents issued, and the connecting lines of different colors represent the cooperative relationship between regions. Compared with the area of other color blocks, the countries represented by red, purple, and light orange blocks have published more articles. These three-color patches represent the United States, Canada, and China. The red block has more connecting lines, indicating that the United States collaborates with other countries more frequently. CiteSpace software forms a cooperative network between countries or regions in Figure 4, in which each node represents a country or region. The purple ring area size indicates the influence of the regional articles, which is equal to centrality. We can see that the United States (101), Canada (54) and China (26) have published the greatest number of articles, and England (0.32), China (0.28), the USA (0.24), Canada (0.22), and Australia (0.16) have strong centrality. The data in Table 1 reflects the aforementioned conclusions. The H-index can accurately reflect academic achievements, the higher the h index, the greater the influence of the paper.

**Table 1:** The top 10 countries or regions with publications on the application of VR in nursing from 2012 to 2021.

**Rank**	**Countries or regions**	**Count**	**Centrality**	**H-index**
1	USA	161	0.24	20
2	Canada	54	0.22	11
3	Australia	26	0.16	7
4	People's Republic of China	23	0.28	7
5	South Korea	21	0.00	7
6	Turkey	15	0.00	4
7	England	15	0.32	7
8	Taiwan	13	0.00	4
9	Spain	11	0.01	4
10	Netherlands	9	0.10	4

### Institutions

**Figure 5:**
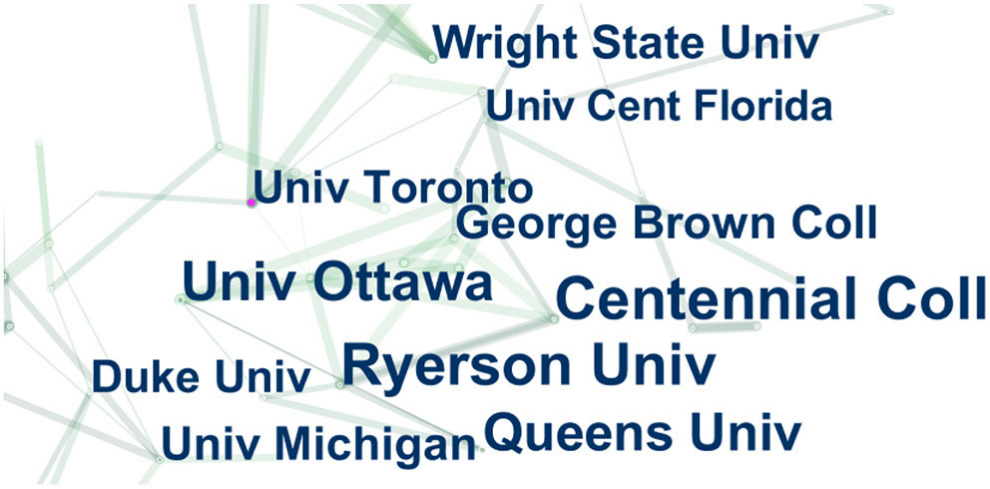
The cooperation of institutions that contributed to publications on applying VR to the study of nursing from 2012 to 2021. Each label in the figure is a research institution. The connecting line between labels indicates the cooperative relationship between institutions.

Table 2 lists the top 10 institutions with the highest number of documents, which are Centennial College (15), Ryerson University (14), University of Ottawa (12), Queen's University Canada (10), Wright State University (8), University of Michigan America (8), George Brown College (8), Duke University (7), University of Toronto Canada (7), University of Central Florida (6). All organizations are located in the United States and Canada. The connecting line between each of the two labels in Figure 5 shows that the institutions in the same country cooperate closely.

**Table 2:** The top 10 institutions with publications on the application of VR in nursing from 2012 to 2021.

**Rank**	**Institutions**	**Country**	**Count**	**H-index**
1	Centennial College	Canada	15	7
2	Ryerson University	Canada	14	6
3	University of Ottawa	Canada	12	4
4	Queen's University Canada	Canada	10	3
5	Wright State University	America	8	6
6	University of Michigan	America	8	4
7	George Brown College	Canada	8	5
8	Duke University	America	7	7
9	University of Toronto	Canada	7	6
10	University of Central Florida	America	6	5

### Journals and Research Category

**Figure 6:**
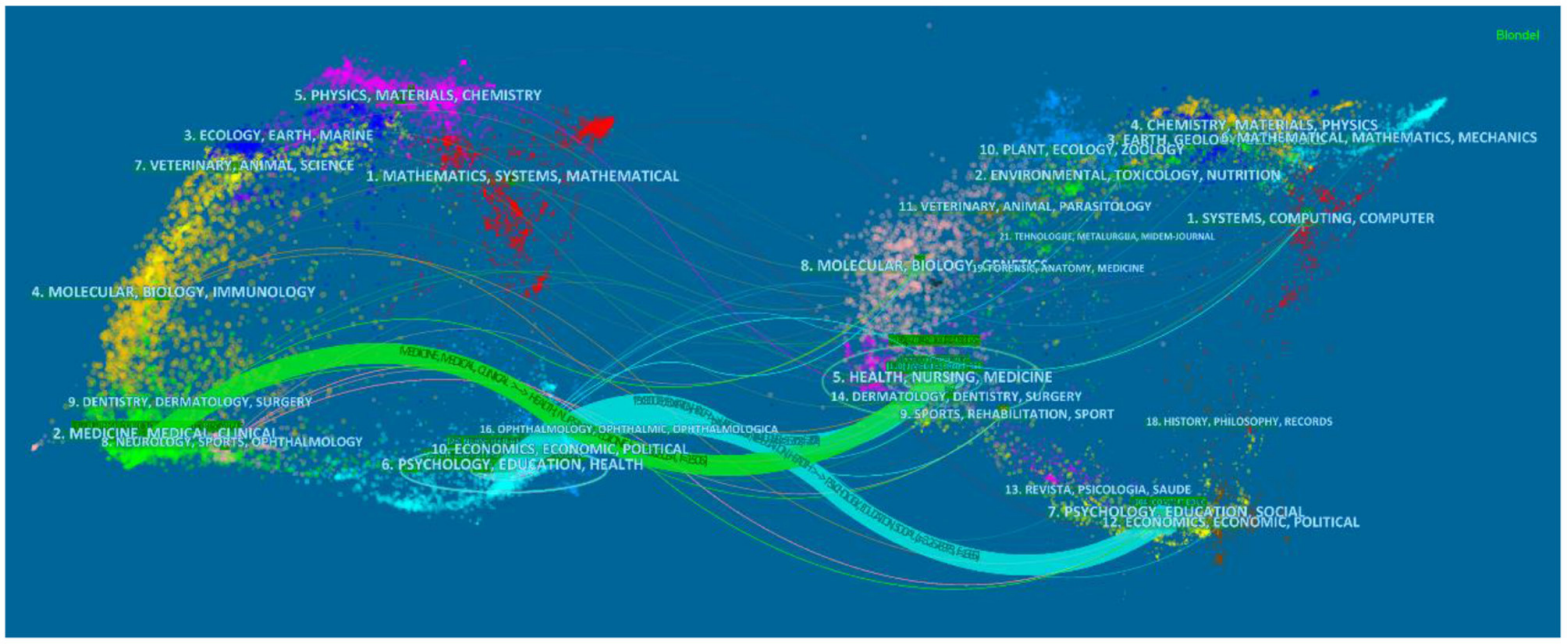
The dual map overlay of journals contributed to publications on the application of VR in Nursing from 2012 to 2021. The green path shows articles in the research fields of MEDICINE / MEDICAL / CLINICAL that are more likely to cite articles in the field of HEALTH / NURSING / MEDICINE. The blue path shows the subject fields of PSYCHOLOGY / EDUCATION / SOCIAL, which are probably cited by PSYCHOLOGY / EDUCATION / HEALTH.

The documents in the cited journals constitute the knowledge base of the citing journal articles. The research fields in high count citing journals constitute a recent research hotspot. Tables 3, 4 show the top 10 citing journals and cited journals, respectively. In terms of research types, the field of medicine/nursing accounts for most of the research in these two journals. At the same time, the research hotspot also includes interdisciplinary application research, including computer science and education. The left and right portions of Figure 6 shows the research field of citing journals and cited journals, respectively. The color curve depicts the citations of journals from various disciplines. The arrow points to the cited publications from various disciplines that are typically referred to by citing journals. The green path shows articles in the research fields of MEDICINE / MEDICAL / CLINICAL that are more likely to cite articles in the field of HEALTH / NURSING / MEDICINE. The blue path shows the subject fields of PSYCHOLOGY / EDUCATION / SOCIAL, which are probably cited by PSYCHOLOGY / EDUCATION / HEALTH. In addition, the picture also shows some research on neurobiology, economics, sports, and ophthalmology in both citing and cited journals.

**Table 3:** The top 10 citing journals of publications on the application of VR in nursing from 2012 to 2021.

**Rank**	**Citing journals**	**Research fields**	**Count**	**2020 Journal impact factor**
1	Clinical Simulation in Nursing	Medicine / Nursing	74	2.391
2	Nurse Education Today	Medicine / Subject education	28	3.442
3	Cin-Computers Informatics Nursing	Computers: Interdisciplinary Applications / Medicine / Nursing	12	1.985
4	Journal of Medical Internet Research	Medicine / Health Care and Services	9	5.428
5	Nurse Educator	Medicine / Nursing	9	2.082
6	Journal of Nursing Education	Medicine / Nursing	8	1.726
7	Nursing Education Perspectives	Medicine / Nursing	7	0.693
8	Journal of Clinical Nursing	Medicine / Nursing	6	3.036
9	Journal of Perianesthesia Nursing	Medicine / Nursing	6	1.084
10	Nurse Education in Practice	Medicine / Nursing	6	2.281

**Table 4:** The top 10 cited journals of publications on the application of VR in nursing from 2012 to 2021.

**Rank**	**Cited journals**	**Research fields**	**Count**	**2020 Journal impact factor**
1	Clinical Simulation in Nursing	Medicine / Nursing	168	2.391
2	Nurse Education Today	Medicine / Subject education	162	3.442
3	Journal of Nursing Education	Medicine / Nursing	98	1.726
4	Nursing Education Perspectives	Medicine / Nursing	94	0.693
5	Journal of the Society for Simulation in Healthcare	Medicine / Health Care and Services	78	1.929
6	Journal of Medical Internet Research	Medicine / Nursing	77	5.428
7	Nurse Educator	Medicine / Nursing	68	2.082
8	Nurse Education in Practice	Medicine / Nursing	66	2.281
9	Journal of Clinical Nursing	Medicine / Nursing	66	3.036
10	Journal of Advanced Nursing	Medicine / Nursing	65	3.187

### Keywords

**Figure 7:**
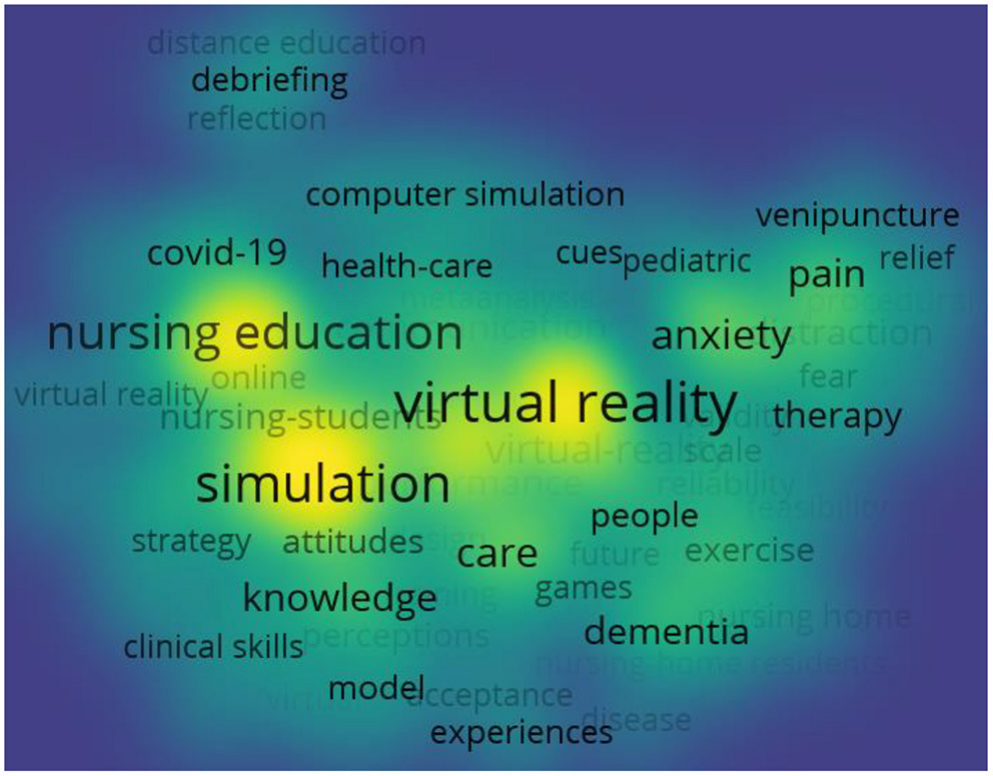
The density visualization of keywords in publications on the application of VR in nursing from 2012 to 2021. A keyword with higher frequency counts forms a yellow region, and those with lower frequency counts form a blue region.

We can analyze the hot keywords of citations using the default setting of the vosviewer software modular clustering algorithm. In Figure 7, the higher the number and frequency of citations, the closer the keyword is to the yellow color block, and the lower they are, the farther it is. Virtual reality, simulation, and nursing education appear to have been the more active keywords used in the research in the past 10 years. At the same time, we use CiteSpace software to analyze the emerging keywords. The default settings parameters were as follows: # Years PerSlice = 2, Top N% = 0.5, pruning algorithm was adopted and minimum duration was 1. Table 5 lists the emergent keywords from the time dimension, which are “virtual reality” (2013–2019), “knowledge” (2020–2021), and “simulation” (2020–2021). These active keywords represent research hotspots.

**Table 5:** Keywords with the strongest citation bursts of publications on the application of VR in nursing from 2012 to 2021.

**Rank**	**Keywords**	**Year**	**Strength**	**Begin**	**End**	**2012–2021**
1	Virtual reality	2012	3.09	2013	2019	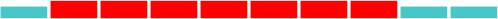
2	Knowledge	2012	3.98	2020	2021	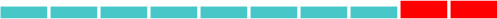
3	Simulation	2012	2.71	2020	2021	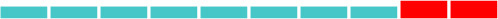

### References

**Figure 8:**
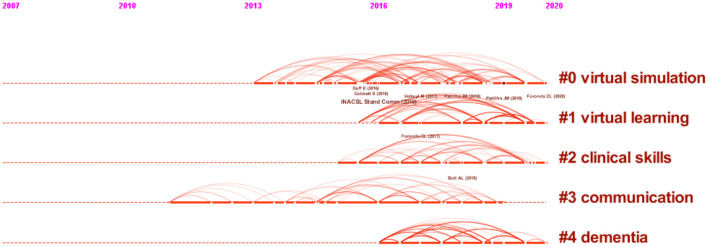
Co-cited references timeline map of publications on the application of VR in nursing from 2012 to 2021. The cited literature is represented by the node on the timeline on the left side of the image. The citation link between documents is represented by curved connecting lines. The clustering label of the co-cited literature may be found on the right side of the image. The stronger the clustering strength, the higher the label position.

We used CiteSpace software with unchanged default settings to analyze the co cited literature. The cited literature constitutes the research basis. The frequency of references represents their influence. The analysis of co-cited documents can show the research theme and development context of a field. Table 6 shows the top 10 cited literature in terms of citation frequency. Among them, two articles supplement and revise the practice standards of the International Nursing Association for clinical simulation and learning. Other literatures, including randomized controlled trials and systematic reviews, all indicate the application of VR as a promising technology in nursing education. The clustering labels of cited documents are obtained from the citing documents. Figure 8 indicates that #0 “Virtual simulation” was the cluster with the widest range. The remaining clusters that have continued into 2020 include #1virtual learning, #2clinical skills, and #4dementia.

**Table 6:** The top 10 references of publications on the application of VR in nursing from 2012 to 2021.

**Rank**	**Title of cited documents**	**DOI**	**Count**	**Interpretation of the findings**
1	INACSL Standards of Best Practice: Simulation^SM^ Simulation Design	doi: 10.1016/j.ecns.2016.09.005	29	This paper lists 11 standards, which supplement and revise the best practice standards of the International Nursing Association for Clinical Simulation and Learning (INACSL): Simulation^SM^.
2	Clinical Virtual Simulation in Nursing Education: Randomized Controlled Trial	doi: 10.2196/11529	20	It is concluded that using the clinical virtual simulator as a resource for the experimental group's education can provide higher learning satisfaction and knowledge improvement than that of the control group after a randomized controlled trial.
3	Virtual vs.Face-to-Face Clinical Simulation in Relation to Student Knowledge, Anxiety, and Self-confidence in Maternal-New Nursing: a Randomized Controlled Trial	doi: 10.1016/j.nedt.2016.08.004	19	This study finds that virtual clinical simulation training may be a promising educational learning tool after assessing the knowledge and self-confidence scores of 56 students who completed a randomized controlled trial.
4	Using Game-Based Virtual Reality with Haptics for Skill Acquisition	doi: 10.1016/j.ecns.2017.09.010	17	The usability score shows that more students are eager to undergo sterile catheterization training using the virtual reality system for skill practice.
5	INACSL Standards of Best Practice: Simulation^SM^ Debriefing	doi: 10.1016/j.ecns.2016.09.008	15	This paper lists 5 standards, which supplement and revise the best practice standards of the International Nursing Association for Clinical Simulation and Learning (INACSL): Simulation^SM^.
6	Virtual Simulation in Nursing Education: A Systematic Review Spanning 1996 to 2018	doi: 10.1097/SIH.0000000000000411	14	This study systematically reviews the research on the impact of virtual simulation on the learning results of nursing students, and puts forward guiding opinions for future research in the field.
7	Virtually Nursing Emerging Technologies in Nursing Education	doi: 10.1097/NNE.0000000000000295	13	This paper introduces six new augmented reality products and systems that can improve nursing education.
8	Online Virtual Simulation and Diagnostic Reasoning: A Scoping Review	doi: 10.1016/j.ecns.2016.04.001	12	In order to prove the effectiveness of online virtual simulation in teaching diagnostic reasoning to health care providers, this study carried out a survey of online classroom virtual simulation contact.
9	Clinical Virtual Simulation in Nursing Education	doi: 10.1016/j.ecns.2017.09.005	12	This study evaluated the simplicity, usefulness and intention of nursing postgraduates in using clinical virtual simulation in nursing research.
10	Virtual Gaming Simulation for Nursing Education: An Experiment	doi: 10.1016/j.ecns.2017.02.004	12	This paper compares students' use of virtual gaming simulation (VGS) with traditional laboratory simulation and points out that the combination of VGS and effective hands-on simulation can become a part of students' teaching and clinical practice.

## Discussion

### Principal Results

The above results show that the number of literatures on the integration of VR and nursing has increased sharply. This is because the COVID-19 pandemic that began in the spring of 2020 hindered many traditional clinics and nursing skill laboratories from providing clinical practice experience for nursing students. Therefore, many nursing students relied on interaction with virtual scenes to progress and complete nursing courses (15). Due to the nursing curriculum proposed by the National Council for State Boards of Nursing in 2016 (16), which recommends the use of simulation as a clinical substitute for traditional clinical experience, research in the field of VR in nursing in the United States has become relatively rich than other countries. In terms of cooperation intensity, Britain, China, the United States, Canada, and Australia have stronger centrality than other countries. It demonstrates that developed countries are the driving force in the medical field and virtual reality in nursing research, but some developing countries are also conducting scientific research. This conclusion is comparable to the national document situation in medical research with virtual reality (17–19). The analysis results of the organization show that the United States and Canada have more research in this field, which is consistent with Garrett, Bernard M (5). Figure 5 shows that there are still a few references among the institutions. However, in Figure 6, the subject fields of both citing and cited journals are very rich, suggesting more room for expansion in this field. At the same time, through keyword co-occurrence and reference cluster analysis, the direction of active topics changing with time is obtained.

### Research Hotspots

#### Burst Keywords

By using VOS viewer and CiteSpace to analyze the similar active keywords, it is calculated that the research hotspots over time are “virtual reality” (2013–2019), “knowledge” (2020–2021) and “simulation” (2020–2021). This shows that research on VR in the nursing industry tends to simulate real scenes in conducting nurse training.

##### Knowledge

Virtual reality nursing related systems transfer health care knowledge, which can be applied in schools, practice bases, and clinical practice. Virtual humans can be built in a virtual world to show anatomical knowledge or develop nurse-patient communication skills (20, 21). Various types of operational training, such as catheterization or tracheal insertion (22, 23), can be realized using the same virtual scene as in an actual laboratory. Generating self-assessment tests as a system that generates self-assessment tests for both teachers and nursing students can also be done through virtual scenes (24, 25). A VR scene can also help new medical and nursing interns by reducing incidences of needle stabbing injury or sharp instrument injury (26). This can benefit new nursing interns who may be afraid of occupational exposure events during practice-based learning. During clinical application, a VR system not only brings convenience to staff, but also actively and effectively interferes with patients' physical and mental health (27, 28).

##### Simulation

Virtual reality can be used to address the situational or economic limitations of traditional education methods that are used to cultivate the skills of medical personnel in dealing with medical and health emergencies. Modern simulation has been developed from the “blue box” in the 1920's (29). In 2013, Daniel Cohen et al. conducted a cohort study with clinicians to determine the feasibility of a low-cost simulated world environment in major event response preparation and training using a virtual pre-hospital bomb explosion scene (30). During the outbreak of COVID-19, VR was used to realize a simulation course to train nurses in dealing with infectious disease disasters. The course included simulated nursing video consultations, a pre-hospital setting, home visits, arrivals at the emergency room, and follow-up rehabilitation home visits (31, 32). In today's community basic health care, which emphasizes humanistic care, nursing training based on a virtual scene is not limited to schools and hospitals, and virtual scenes for community and family nursing are constantly being developed. Yvonne l et al. proved that virtual nursing can effectively cultivate students to cross geographical barriers, acquire multiculturalism, and enhance their cultural ability through a prenatal/postpartum virtual simulation experience of African American and Amish patients (33).

In general, the use of virtual nursing in training nursing students shows promise. Many virtual scenes, such as first aid for disaster accidents, invasive operations, and nursing care models that inject cultural differences, need to be developed by staff.

#### Clusters of References

Highly cited references affect the frontier development of research, mainly in the field of nursing education, which is consistent with the analysis results of hot keywords. The clustering of citations continue until the most recent references can predict the research trends, which are virtual simulation, virtual learning, clinical skills, and dementia. We can find that dementia has become a hot research keyword.

##### Virtual Clinical Learning Simulation

The largest clustering result of citations contains virtual simulation, virtual learning, and clinical skills. This means that various virtual reality systems suitable for nurse training are being developed.

The use of high fidelity simulation technology has been suggested as a means of providing clinical experience for nursing students (34). The purpose of virtual clinical simulation (VCS) in nursing, which can allow one or more people to participate, is to cultivate students' professional skills (35). Since the behavior intention of interns must be understood in the development of VCS (36), various virtual clinical skill training systems have been continuously developed and improved. The virtual nursing system promoting humanistic care covers, but is not limited to, the following: nursing various organs in the human body, mental and psychological care (37), oral nursing (38) skills in knee arthroplasty (39), hysteroscopic surgery nursing (40), nasogastric tube placement (41), and surgical suture (42). The nurse training program also includes management ability is (43). It is also worth mentioning that VR has helped to realize telemedicine services (44).

Virtual nursing training is not only an optional teaching modality in different nursing operations, but it also breaks down regional and spatial barriers and introduces new ideas to telemedicine service development.

##### Dementia

Another result of the clustering of citations shows dementia as a research trend. In 2018, 50 million people worldwide suffered from dementia, an increase of 6% from 2015 (45). Due to the aggravation of the aging problem of the global population, dementia is becoming more and more prominent. However, the lack and uneven distribution of existing medical resources has not yet been resolved. This phenomenon forces researchers to develop intervention scenarios for virtual treatment that are more scientific.

Virtual scene training for dementia allows for the participation of both medical staff and patients. In order to improve nurses' empathy for dementia patients and help improve the living environment of dementia patients, Paul Slater et al. and Jennifer Stargatt et al. discussed using a VR project as a tool to help health care professionals sympathize with dementia patients (46–48). Patients with dementia have cognitive impairment and various behavioral and psychological symptoms, such as cognitive disorder, tension, depression, psychosis, shouting, and violence (49). Wendy Moyle et al. measured and described the effectiveness of participation, indifference and the emotional state of dementia patients through a VR forest, and concluded that virtual technology has a positive impact on them (50). Jorge Oliveira et al. reported a pilot randomized controlled trial involving 17 subjects and explored the effect of cognitive stimulation reproducing several instrumental activities of daily life in VR on patients with dementia caused by mild to moderate Alzheimer's disease. They concluded that VR is helpful in maintaining the cognitive function of patients with Alzheimer's disease. Jung-Hee Kim et al. recruited 10 Korean dementia patients and developed a VR intervention program based on their psychological needs. The scheme was proven to be convenient and safe as the program alleviated the patients' behavioral and psychological symptoms (51).

Developing VR scenes for dementia can help nurses and patients make positive adjustments for abnormal behavior and psychology. The construction of a VR scene should refer to the physical and psychological needs of different groups, such as different races, genders, and lifestyles, to improve comfort of use. There is still great room for development in this field.

#### Open Challenges and Future Opportunities in Virtually Nursing

The research fields of citation journals are rich, covering medicine, rehabilitation, neurobiology, psychology, economics, kinematics, computer science, and mathematics. This shows that the development of today's VR in the field of nursing is inseparable from the basic knowledge of various disciplines.

In recent years, there has been a growing demand for geometric problems in emerging application fields, such as VR. Bogucka et al. introduced the modeling and processing of geometric data, which still faces problems that need to be solved (52). In programming, a deep understanding of software code is required. A VR digital gamification method for program code, which applies digital gamification to a multi metaphor VR visualization of software program structure was described and evaluated by Oberhauser et al. The results of their preliminary empirical investigation described and evaluated a VR digital gamification method for program code, which applies digital gamification to multi metaphor VR visualization of software program structure. The results of their preliminary empirical investigation show that it is possible to increase subjects' fun and motivation as well as focus attention, and encourage the exploration of software structure (53). Since the development of the new system requires labor costs, it is necessary to ensure the practicability and effectiveness of the scene. In a study on the use of VR therapy for pain patients in 2018, it was found that VR in a certain range can help patients and hospitals save costs. However, going beyond this range may result in economic burdens to the hospital (54). In a 2021 survey, Roman et al. found that VR provided an alternative tourism model during the COVID-19 pandemic. At the same time, a VR tourism environment can simulate safe travel by allowing users to visit certain destinations despite political restrictions and economic difficulties. Moreover, VR technology can help people meet their spiritual and psychological needs during some special periods. For example, it can reduce the anxiety of mothers during childbirth and the fear of children undergoing surgery (6, 28, 55). In the past 2 years, more personalized and refined VR with neurotic feedback systems have been developed. Tartarisco, G et al. trained 20 nurses to simulate workplace stress situations. A neuro fuzzy model was used to collect the subjects' heart rate, breathing, and activity during training. The model was found to show good performance in the classification of stress level (56).

In summary, there is still a very broad space for the development of VR in the field of nursing. For nurse training, more high-risk or disaster first aid scenes need to be developed. To meet the needs of special groups, a theoretical framework to support the construction of virtual scenes must be used to ensure the effectiveness of the system. The introduction of biological signal feedback model is also a good idea. Economic and political factors deserve to be included in the system design plan.

### Limitations

There are some potential limitations to this study. To begin, we only looked at published literature from 2012 to 2021. Some studies are ongoing but have not yet been published. Second, we only searched the literature in the woscc database, a popular academic database. There may be minor differences if you include citations from other databases, such as Google Scholar or PubMed. However, because different database citation counting methods differ, fusing and analyzing at the same time is impossible. Third, even though we read the 408 articles analyzed in this paper at the same time, we cannot rule out the researchers' inherent bias.

## Conclusion

Nursing VR products have been used or are being used in many countries, but the number of SCI research papers in this area is not very large. As a new field in nursing research, virtual nursing has had an impact on both nurses and clients. It covers a wide range of disciplines and has applications in the full lifecycle. Nursing research tends to focus on nurse education and the elderly. The design of virtual scenes can be edited with reference to the dynamic standards of the International Nursing Association for Clinical Simulation and Learning (INACSL). Virtual scenes need to be tested before they are put to use in order to get a suitable and effective virtual training system.

Today's health care system is complex, and nursing is becoming more and more professional. Nursing students must be prepared for emergency and basic community nursing environment. Based on the above factors, the research and application of nursing VR have become more extensive. Given the COVID-19 pandemic, more VR scenes need to be designed and applied to staff training and home isolation staff care. With the emergence of the concept of the metaverse, the research and application of VR technology in nursing will gradually increase and the links between various disciplines in this field will become closer.

## Data Availability Statement

The original contributions presented in the study are included in the article/supplementary material, further inquiries can be directed to the corresponding authors.

## Author Contributions

JZ, YL, and FZ acquired, analyzed the data, and drafted the manuscript. RM and FF designed the research, acquired the article information, and revised the manuscript. All authors contributed to the article and approved the submitted version.

## Funding

The work was financially supported in part by the Zhejiang Medical and Health Research Project (2020PY027), and the Huzhou Science and Technology Planning Program (2020GY44), Key R&D and Promotion Projects in Henan Province (222102310615).

## Conflict of Interest

The authors declare that the research was conducted in the absence of any commercial or financial relationships that could be construed as a potential conflict of interest.

## Publisher's Note

All claims expressed in this article are solely those of the authors and do not necessarily represent those of their affiliated organizations, or those of the publisher, the editors and the reviewers. Any product that may be evaluated in this article, or claim that may be made by its manufacturer, is not guaranteed or endorsed by the publisher.
